# Selective Disruption of Aurora C Kinase Reveals Distinct Functions from Aurora B Kinase during Meiosis in Mouse Oocytes

**DOI:** 10.1371/journal.pgen.1004194

**Published:** 2014-02-27

**Authors:** Ahmed Z. Balboula, Karen Schindler

**Affiliations:** 1 Department of Genetics, Rutgers, The State University of New Jersey, Piscataway, New Jersey, United States of America; 2 Theriogenology Department, Faculty of Veterinary Medicine, Mansoura University, Mansoura, Egypt; University of Edinburgh, United Kingdom

## Abstract

Aurora B kinase (AURKB) is the catalytic subunit of the chromosomal passenger complex (CPC), an essential regulator of chromosome segregation. In mitosis, the CPC is required to regulate kinetochore microtubule (K-MT) attachments, the spindle assembly checkpoint, and cytokinesis. Germ cells express an AURKB homolog, AURKC, which can also function in the CPC. Separation of AURKB and AURKC function during meiosis in oocytes by conventional approaches has not been successful. Therefore, the meiotic function of AURKC is still not fully understood. Here, we describe an ATP-binding-pocket-AURKC mutant, that when expressed in mouse oocytes specifically perturbs AURKC-CPC and not AURKB-CPC function. Using this mutant we show for the first time that AURKC has functions that do not overlap with AURKB. These functions include regulating localized CPC activity and regulating chromosome alignment and K-MT attachments at metaphase of meiosis I (Met I). We find that AURKC-CPC is not the sole CPC complex that regulates the spindle assembly checkpoint in meiosis, and as a result most AURKC-perturbed oocytes arrest at Met I. A small subset of oocytes do proceed through cytokinesis normally, suggesting that AURKC-CPC is not the sole CPC complex during telophase I. But, the resulting eggs are aneuploid, indicating that AURKC is a critical regulator of meiotic chromosome segregation in female gametes. Taken together, these data suggest that mammalian oocytes contain AURKC to efficiently execute meiosis I and ensure high-quality eggs necessary for sexual reproduction.

## Author Summary

Precise control of chromosome segregation is essential for generating cells with the proper number of chromosomes. In germ cells, sperm and egg, an abnormal chromosome number leads to infertility, miscarriage, or, in the case of a live birth, developmental disorders such as Down Syndrome. For reasons not entirely clear, eggs are more prone to chromosome segregation mistakes than sperm. In this study, we study the role of a regulator of chromosome segregation, Aurora C kinase, in mouse oocytes. This is the first study to separate its function from Aurora B kinase that is highly similar in sequence. We find Aurora C is uniquely required to produce eggs with the proper number of chromosomes.

## Introduction

Haploid gametes are generated by meiosis, a unique cell division process that consists of a single round of DNA replication followed by two successive cell divisions. In the first division, meiosis I (MI), homologous chromosomes segregate. The second division, meiosis II (MII), is more similar to mitosis because sister chromatids segregate. An error in chromosome segregation can result in aneuploidy, the leading genetic cause of infertility and congenital birth defects in humans [1], [2]. It is now well appreciated that the incidence of aneuploidy is at least 10-fold higher in female gametes (oocytes) than it is in male gametes (sperm) [3]. Thus, understanding the underlying causes of oocyte aneuploidy could help address a majority of clinical aneuploidies in humans.

During meiosis there are a number of possible mistakes that could result in aneuploidy. These mistakes include, but are not limited to, defects in kinetochore-microtubule (K-MT) attachments, a faulty spindle assembly checkpoint (SAC), improper cytokinesis, or loss of sister chromatid cohesion [4]–[10]. In mitosis, the chromosomal passenger complex (CPC) is essential for steering the chromosomes through these obstacles [11]–[16]. The CPC does this through a sophisticated pattern of synchronized movements. At metaphase, the CPC localizes to kinetochores, and at anaphase, it relocates to the spindle midzone. This dynamic localization pattern ensures that the CPC phosphorylates the right substrates at the right time and place. Perturbing the CPC in oocytes often leads to errors in MI, thereby resulting in aneuploidy [6], [17].

In mitotically dividing cells, the CPC consists of a catalytic subunit, Aurora B kinase (AURKB), and regulatory subunits Inner Centromere Protein (INCENP), Survivin, and Borealin [18]–[20]. Meiotic cells, however, contain another enzymatic subunit, Aurora C kinase (AURKC), that can function in the CPC in place of AURKB [6], [21]–[25]. AURKB and AURKC are members of a conserved serine-threonine protein kinase family, and are highly similar in sequence within their catalytic domains. Both AURKs bind the IN box region of INCENP, but not at the same time [22]. This binding is essential to stimulate kinase activity and for subsequent phosphorylation of INCENP [26]. Because they are highly similar in sequence, AURKC can compensate for loss of AURKB when ectopically expressed in somatic cells and supports mitosis in preimplantation mouse embryos that lack AURKB [27]–[29]. Furthermore, AURKB compensates for the loss of AURKC in oocytes from *Aurkc^−/−^* mice [23].

The sequence similarities between AURKB and AURKC have hindered our understanding of their functions during meiosis. For example, small molecule inhibitors do not selectively inhibit the kinases [17], [22], [24], [25], [30], siRNA knockdown approaches are inefficient and lack specificity [17], [22], and, as mentioned, genetic knockout strategies allow for functional compensation [23], [29]. We hypothesized that expression of a dominant negative allele of AURKC (AURKC-DN) perturbed both AURKB and AURKC functions in oocytes [6]. To further understand the molecular mechanisms that lead to the high incidence of aneuploidy in mammalian oocytes, we sought to develop a tool to selectively disrupt AURKC function.

Mutation of the gatekeeper leucine residue in the ATP-binding pocket of AURKB inactivates the kinase *in vivo* and *in vitro* [31]. Here, we devised a similar strategy to inhibit AURKC activity and demonstrate that an AURKC-L93A gatekeeper mutant selectively disrupts AURKC, but not AURKB, function during oocyte meiosis. Using this strategy, we show that AURKC has non-overlapping functions with AURKB during MI. We find that loss of AURKC function results in misalignment of chromosomes and arrest at metaphase I (Met I), and, in oocytes that failed to arrest, aneuploidy at metaphase II (Met II). Oocytes expressing the AURKC mutant failed to correct erroneous K-MT attachments, which is the likely cause of the misaligned chromosomes and aneuploidy. We also find that AURKC-CPC is not uniquely required to maintain an active SAC or to execute cytokinesis. These events may be AURKB-CPC specific or require the activity of both AURKB and AURKC. This study is the first to ascribe non-overlapping functions of AURKC from AURKB during MI in mouse oocytes.

## Results

### Dominant negative AURKC disrupts both AURKB and AURKC function during oocyte meiosis

**Figure 1:**
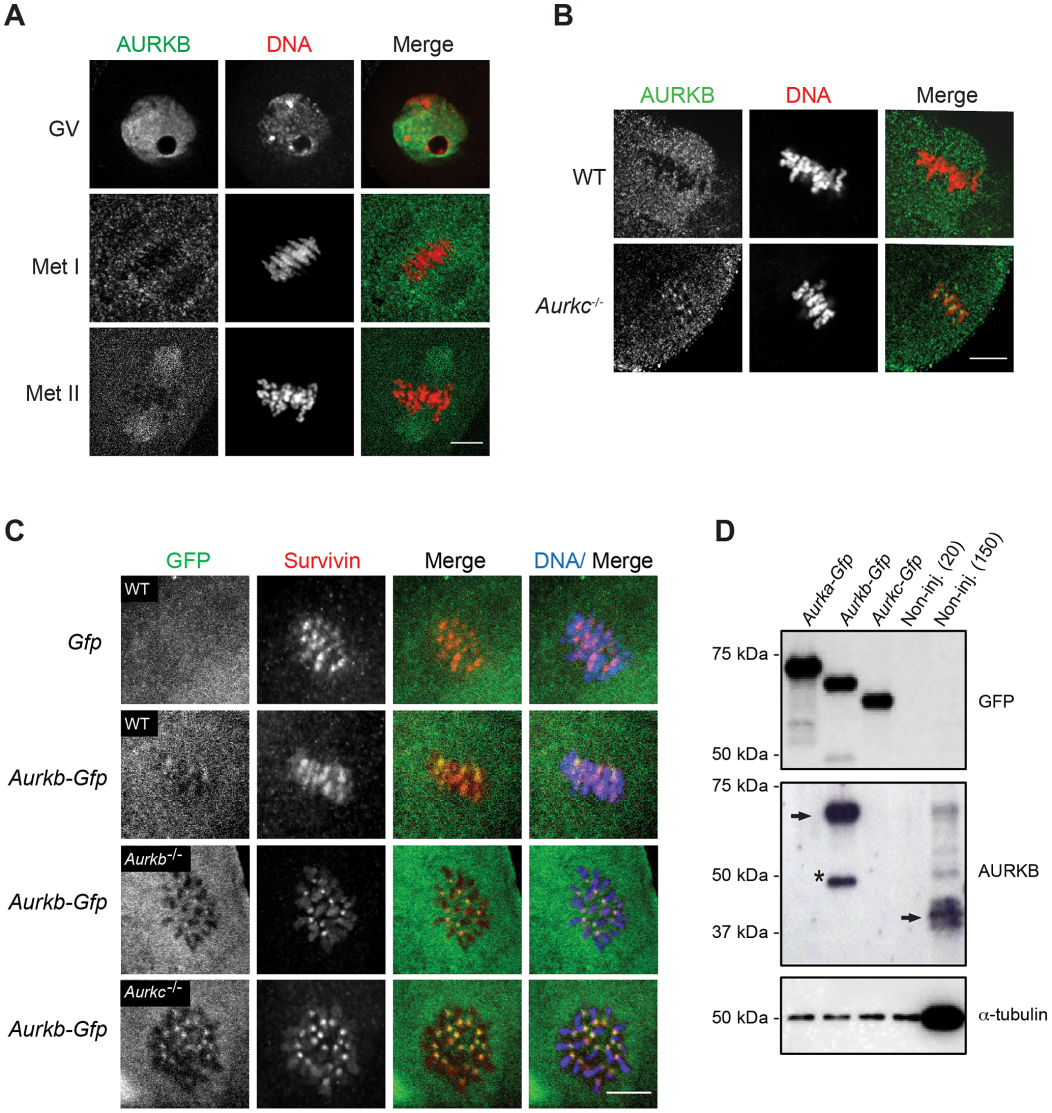
AURKB is expressed in mouse oocytes. (A) GV-intact oocytes were collected from CF1 mice and matured *in vitro* for 8 h (Met I), or 16 h (Met II), prior to fixation and staining with an anti-AURKB antibody. (B) GV-intact oocytes were collected from WT and *Aurkc^−/−^* mice and matured *in vitro* for 16 h (Met II), prior to fixation and staining with an anti-AURKB antibody. Merged images show AURKB in green and DNA in red. (C) GV-intact oocytes were collected from WT, *Aurkb* ^−/−^, and *Aurkc^−/−^* mice, microinjected with the indicated cRNA, and matured *in vitro* for 16 h (Met II), prior to fixation and staining with an anti-Survivin antibody. Merged images show AURKB-GFP in green, Survivin in red, and DNA in blue. These experiments were conducted with a minimum of 20 oocytes for each group. Shown are representative images (Scale bar, 10 µm). (D) 20 GV-intact oocytes were collected from CF1 mice and microinjected with the indicated cRNA. Two hours after injection, the oocytes were matured to Met II *in vitro* (16 h). The total numbers of non-injected control oocytes (Non-inj.) are indicated in parenthesis. Total cellular lysates were probed with the indicated antibody. The panels are images of the same membrane that was stripped and re-probed. The arrows indicate the specific AURKB protein band, and the asterisk indicates a presumed degradation product of AURKB-GFP.

Expression of dominant negative AURKC (AURKC-T171A, T175A) (referred hereafter as AURKC-DN) in mouse oocytes causes cytokinesis failure and misaligned univalent chromosomes at MI ([6] and Figure S1). These phenotypes are identical to that of oocytes cultured in high concentrations of small molecule inhibitors of AURKB (ZM447439 and AZD1152) which likely also inhibit AURKC [17], [25], [32]. Two models could explain the similarity in phenotype between the two perturbations: 1) Either AURKB is not expressed in mouse oocytes or 2) AURKC-DN disrupts both AURKB and AURKC function. To investigate the first model, we assessed the protein expression of AURKB in oocytes undergoing meiosis via immunocytochemistry with an antibody previously validated to detect AURKB in mouse preimplantation embryos [29]. AURKB localized within the nucleus of prophase-arrested oocytes and with the meiotic spindle at Met I and II (Figure 1A). This localization pattern is different than that of AURKC, which localizes to kinetochores and the Met I inter-chromatid axis [23], [24]. To further confirm the specificity of the antibody, we examined AURKB in oocytes from *Aurkc^−/−^* mice. In oocytes from WT littermates, AURKB localized to the meiotic spindle, but in *Aurkc^−/−^* oocytes at Met II AURKB localized to kinetochores (Figure 1B). Exogenous AURKB-GFP also localized to kinetochores in *Aurkb* ^−/−^ and *Aurkc^−/−^* oocytes at Met II, as previously demonstrated [23] where it co-localized with Survivin, a CPC subunit (Figure 1C). In addition, this co-localization occurred at Met I (Figure S2). Similar to overexpression of AURKB-GFP in WT oocytes, we still detect AURKB-GFP in the spindle region in the single knockout oocytes (Figure S2). We also assessed the specificity of the antibody by immunoblot analysis. We microinjected wild-type oocytes with cRNAs encoding *Aurka-Gfp*, *Aurkb-Gfp*, or *Aurkc-Gfp*. Probing with anti-GFP antibody, confirmed expression in each group (Figure 1D, top panel). When we probed the membrane with the anti-AURKB antibody, it cross-reacted only with oocytes injected with *Aurkb-Gfp* (Figure 1D, middle panel). We, and others, previously demonstrated the presence of *Aurkb* mRNA in oocytes [6], [23]–[25], and another report documents AURKB protein in mouse oocytes [33]. Furthermore, when we probed pooled whole-cell lysates from 150 non-injected oocytes, we detected a band at ∼40 kDa, which is the expected size of endogenous AURKB (Figure 1D, middle panel, right-most lane). Taken together, these data support the model that AURKB is expressed in mouse oocytes. Furthermore, the localization of endogenous AURKB at kinetochores in oocytes from *Aurkc^−/−^* mice supports the hypotheses that either AURKB compensates for the loss of AURKC [23] and/or endogenous AURKC competes with AURKB for localization at kinetochores and the inter-chromatid axis.

**Figure 2:**
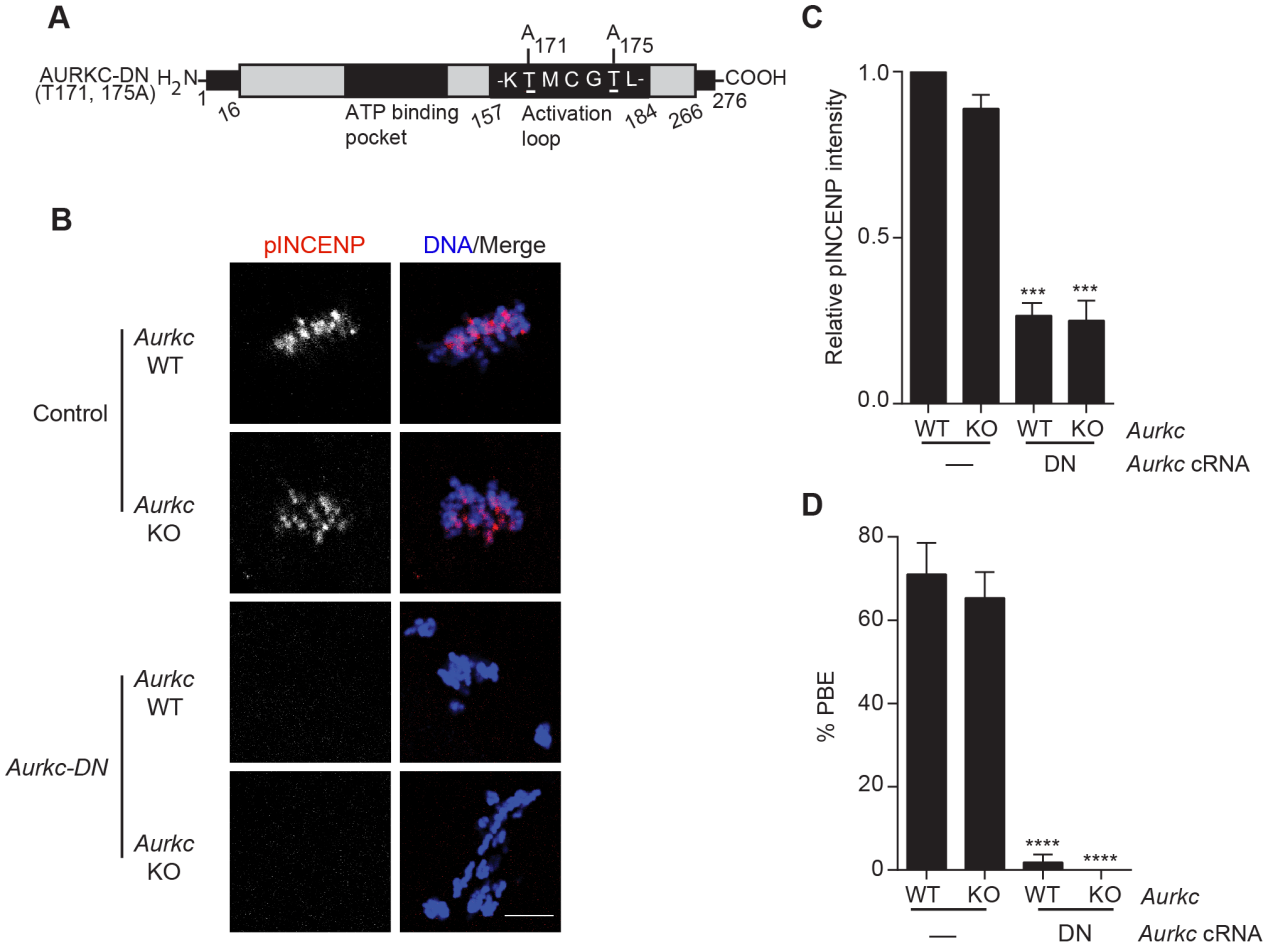
Dominant-negative AURKC (AURKC-DN) disrupts both AURKB/C function in oocytes. (A) Schematic representation of AURKC-DN. The mutated threonines (T) in the activation loop are underlined. (B–D) Full-grown WT (*Aurkc* WT) and *Aurkc^−/−^* (*Aurkc* KO) oocytes were injected with PBS or *Gfp* (Control) or *Aurkc-DN* cRNA. The injected oocytes were matured for 16 h, followed by fixation and immunostaining with a phospho-specific INCENP (pINCENP) antibody (red in merge). DNA was detected via DAPI staining (blue). Shown are representative confocal Z-projections (scale bar, 10 µm). (C) Corresponding quantification of pINCENP pixel intensities in B. This experiment was conducted 3 times with a minimum of 20 oocytes in each group. (D) Percentage of oocytes that extruded polar bodies (PBE). One-way ANOVA was used to analyze the data. *** P<0.001, **** P<0.0001.

To investigate whether AURKC-DN (Figure 2A) disrupts both AURKB and AURKC function in oocyte meiosis, we eliminated issues with redundancies by using oocytes from *Aurkc* ^−/−^ mice (i.e. containing only AURKB) [23]. As anticipated, microinjection of WT or *Aurkc* ^−/−^ oocytes with *Aurkc-DN* cRNA resulted in the same phenotypes. These oocytes did not have detectable phosphorylated INCENP (pINCENP) (Figure 2B–C). They contained misaligned univalent chromosomes, and they failed to divide, as examined by polar body (PB) extrusion (Figure 2B, D). These data support the second model: AURKC-DN perturbs the function of both AURKB and AURKC, making it inadequate for assigning specific function to AURKC.

### A gatekeeper AURKC mutant is catalytically inactive and selectively disrupts AURKC

**Figure 3:**
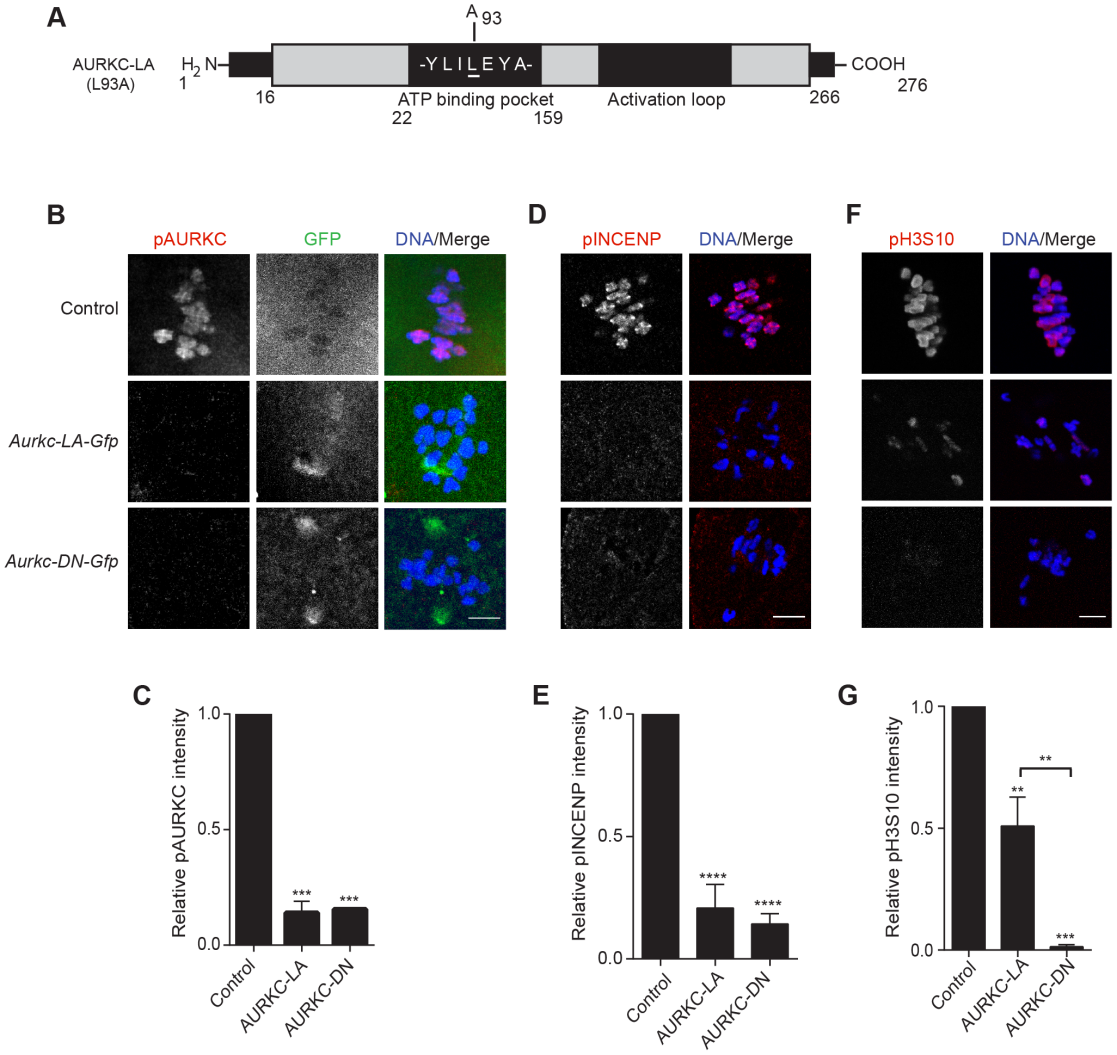
AURKC-LA and AURKC-DN are catalytically inactive. (A) Schematic representation of AURKC-LA. The mutated leucine (L) residue in the ATP binding pocket is underlined. (B–G) Full-grown WT oocytes were injected with the indicated cRNA; controls were injected with PBS or *Gfp* cRNA. Met I oocytes were fixed and examined for phosphorylated AURKC (pAURKC) (red in merge) and GFP expression (green in merge) (B), phosphorylated INCENP (pINCENP) (red in merge) (D) and phosphorylated H3S10 (pH3S10) (red in merge) (F). DNA was detected via DAPI staining (blue). Shown are representative Z-projections from confocal microscopy (scale bars, 10 µm). (C, E, and G) Corresponding quantification of fluorescence intensity of B, D, and F, respectively. The experiment was conducted at least 2 times with a minimum of 20 oocytes in each group. Shown are representative images. One-way ANOVA was used to analyze the data. **P<0.01; *** P<0.001; **** P<0.0001.

Because AURKC-DN disrupts the function of both AURKB and AURKC, and because AURKB compensates for loss of AURKC [23], we sought to develop a tool to selectively perturb AURKC function. The dominant negative mutation involves changing two threonines in the activation loop to non-phosphorylatable alanine residues [6], [34] (Figure 2A). Therefore, this mutant, while not activated, can presumably bind ATP and substrates. Protein kinases are commonly bi-lobed in structure thereby generating a pocket for ATP binding and catalysis. Within the ATP binding pocket are conserved “gatekeeper” residues that restrict binding of other intracellular molecules [35], [36]. Mutation of a gatekeeper residue to an alanine enlarges the pocket, but generally does not perturb the function of most protein kinases. However, approximately 30% of kinases do not tolerate a mutation of gatekeeper residues [31], [35], [36]. Mutation of the gatekeeper residue (L159) in murine AURKB renders the kinase inactive, and it is unable to phosphorylate histone H3 in mitotic cell extracts [31]. Based on protein sequence alignment, we determined that the gatekeeper residue in AURKC is L93 (Figure 3A). Because mutation at this residue likely affects ATP binding instead of activation, we postulated that mutating L93 to A (hereafter referred to as AURKC-LA) might behave differently than the dominant negative and could selectively disrupt AURKC. To first confirm that AURKC-LA is not active, we microinjected WT oocytes with *Aurkc-LA-Gfp* cRNA. We assessed activity by immunostaining the injected oocytes with phospho-specific antibodies that recognize AURKB/C substrates. We found loss of auto-phosphorylated AURKC activation signal (pAURKC; pT171) and significantly decreased pINCENP (pS893/S894) compared to control injected oocytes (Figure 3B–E). Compared to controls, phosphorylated histone H3 (pH3S10) was reduced by ∼50% in LA-injected oocytes (Figure 3F–G). The levels of pAURKC and pINCENP were reduced almost to the same levels as they were in *Aurkc-DN* injected oocytes (Figure 3B, D). On the other hand, pH3S10 signals were completely inhibited only in the AURKC-DN oocytes, suggesting that H3S10 is a target of both AURKB and AURKC (Figure 3F–G). We note that phosphorylation of H3S10 in mitotic cells is less sensitive to localized AURKB activity compared to other substrates [37], and our data is consistent with this observation. These data suggest that AURKC-LA is catalytically inactive, and that it inhibits endogenous AURK/CPC activity.

**Figure 4:**
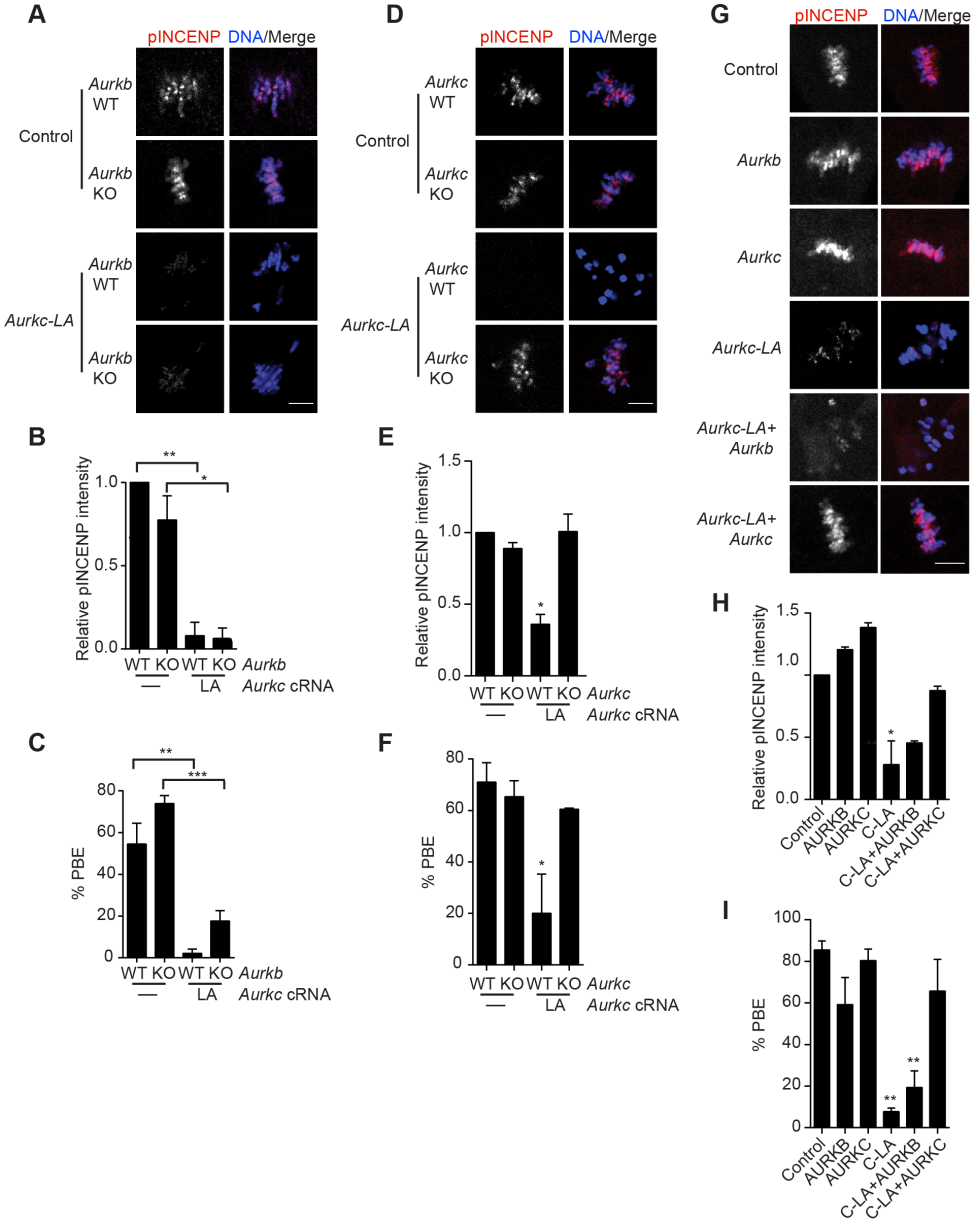
AURKC-L93A (AURKC-LA) is catalytically inactive, and selectively disrupts AURKC function. (A–I) Full-grown WT or *Aurkb* ^−/−^ (A–C), WT or *Aurkc* ^−/−^ oocytes (D–F) or WT CF1 oocytes (G–I) were injected with the indicated cRNA; controls were injected with either PBS or *Gfp* cRNA. The microinjected oocytes were matured *in vitro* to Met II (16 h) followed by pINCENP detection (red in merge) via confocal microscopy. DNA was detected by DAPI staining (blue). Shown are representative Z-projections (scale bar, 10 µm). (B, E, H) Corresponding quantification of pINCENP intensities. (C, F, I) Percentage of oocytes that extruded polar bodies (PBE). The experiments were conducted 3 times with a minimum of 15 oocytes in each group. One-way ANOVA was used to analyze the data. * P<0.05, ** P<0.01, *** P<0.001.

To test our hypothesis that the gatekeeper mutant specifically inhibits AURKC, we first microinjected *Aurkc-LA* in *Aurkb* ^−/−^ oocytes (which express only AURKC; Figure 4A–C) and in *Aurkc* ^−/−^ oocytes (which express only AURKB, and that compensates for AURKC [23]; Figure 4D–F). Control mice were WT littermates from each genetic background. AURKC-LA significantly reduced INCENP phosphorylation and PB emission in *Aurkb* ^−/−^ oocytes suggesting that the catalytically inactive AURKC-LA efficiently disrupts endogenous AURKC function (Figure 4A–C). Importantly, *Aurkc* ^−/−^ oocytes expressing AURKC-LA extruded PBs and had normal levels of phosphorylated INCENP, similar to WT and KO injected controls (Figure 4D–F). These data confirm that AURKC-LA selectively disrupts AURKC function without disrupting endogenous AURKB function.

To further test the specificity of AURKC-LA, we conducted a rescue experiment in WT oocytes. Co-expression of WT AURKC rescued the AURKC-LA phenotypes. INCENP was phosphorylated to near control levels and PBs were extruded. Co-expression of WT AURKB did not rescue the phenotypes (Figure 4G–I). These data further confirm that AURKC-LA does not perturb AURKB function and suggests that the defect in INCENP phosphorylation and block in meiotic maturation are specific for loss of AURKC function.

### AURKC is required to retain CPC localization during MI of mouse oocytes

**Figure 5:**
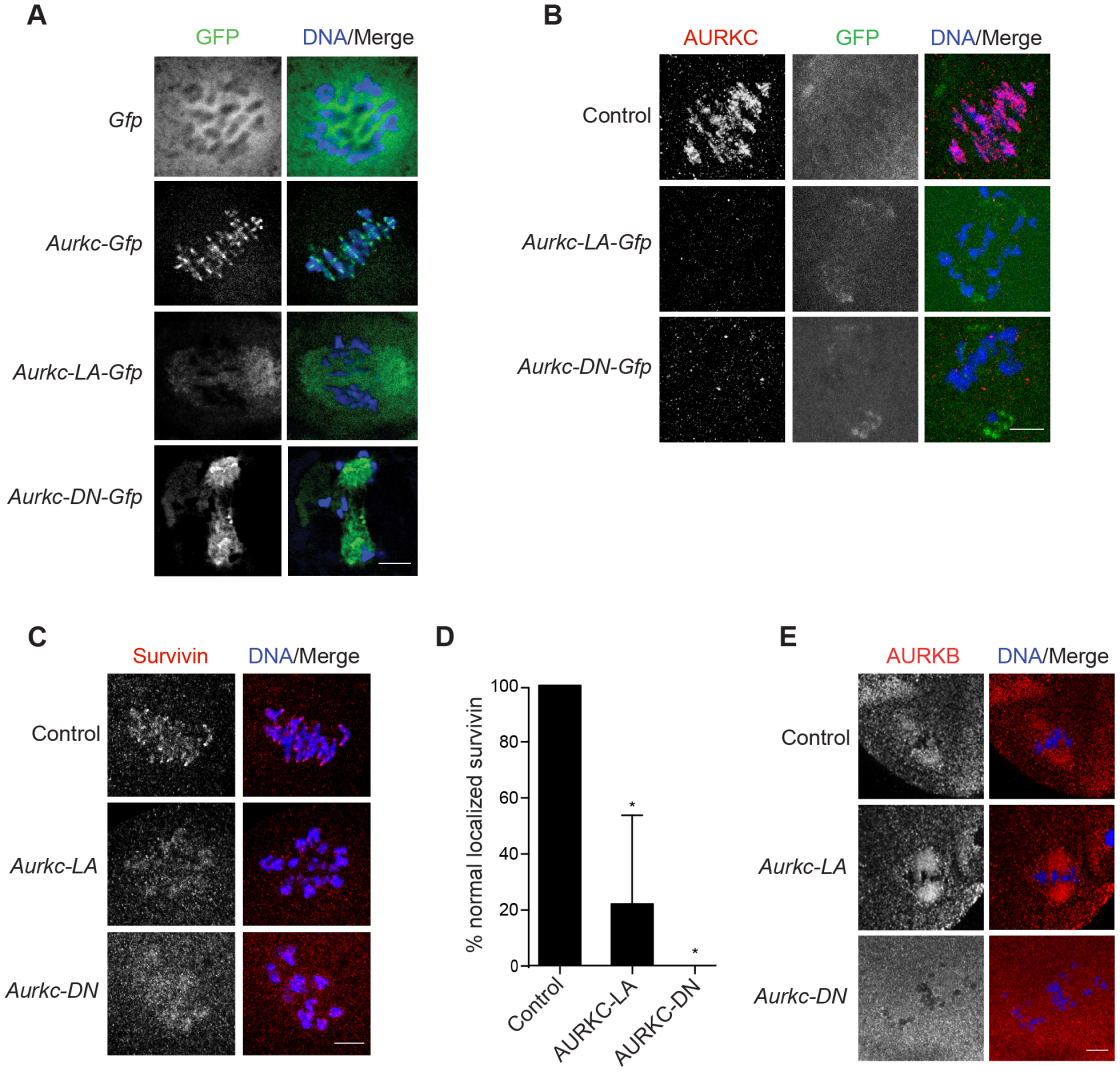
AURKC is required to retain CPC localization at Met I. Full-grown oocytes were injected with the indicated cRNA; controls were injected with PBS or *Gfp* cRNA. After 8 h of *in vitro* maturation, Met I oocytes were fixed and examined for localization of the GFP-tagged mutant protein (green in merge) (A), endogenous AURKC (red in merge) (B), endogenous Survivin (red in merge) (C), and endogenous AURKB (red in merge) (E). DNA was detected via DAPI staining (blue). Shown are representative confocal Z-projections (scale bars, 10 µm). (D) Corresponding quantification of oocytes with properly localized Survivin in B. The experiments were conducted at least 2 times with a minimum of 20 oocytes in each group. One-way ANOVA was used to analyze the data. *P<0.05.

In budding yeast, aurora kinase activity is required for proper CPC localization and prevents premature localization of the CPC to the spindle [38]. In mitotically dividing tissue culture cell lines, inactive AURKB mutants fail to localize normally at centromeres [31], [39]. To investigate if AURKC-LA behaves similar to WT AURKC, we analyzed its subcellular localization in oocytes at Met I. WT AURKC localized to kinetochores and inter-chromatid axes of Met I oocytes (Figure 5A) as previously reported [23], [24]. On the other hand, AURKC-LA and AURKC-DN failed to localize normally. Both mutants localized predominantly with the spindle. Therefore, AURKC activity may be required to regulate CPC localization.

To examine the changes in CPC localization, we first assessed the localization of endogenous AURKC. When oocytes expressed either AURKC-LA or AURKC-DN we could not detect endogenous AURKC on the chromosomes (Figure 5B). For reasons not determined, we note that the antibody used to detect AURKC on chromosomes is not compatible with detecting de-localized AURKC-LA. Survivin is also a member of the CPC, and is expressed during mouse oocyte meiosis [40], [41]. Similar to AURKC-DN, oocytes expressing AURKC-LA resulted in displacement of the CPC at Met I as evidenced by the loss of kinetochore and inter-chromatid axis localization of endogenous Survivin (Figure 5C–D). Importantly, AURKC-LA did not alter the spindle localization of AURKB as compared to AURKC-DN, further supporting our evidence that AURKC-LA selectively perturbs AURKC (Figure 5E). These findings are consistent with previous observations that loss of AURKB/C kinase activity by using small molecule inhibitors results in displacement and atypical localization of the CPC in mitosis [31], [42] and oocyte meiosis (our unpublished observations).

### Efficient meiotic progression and chromosome alignment requires AURKC activity during MI

**Figure 6:**
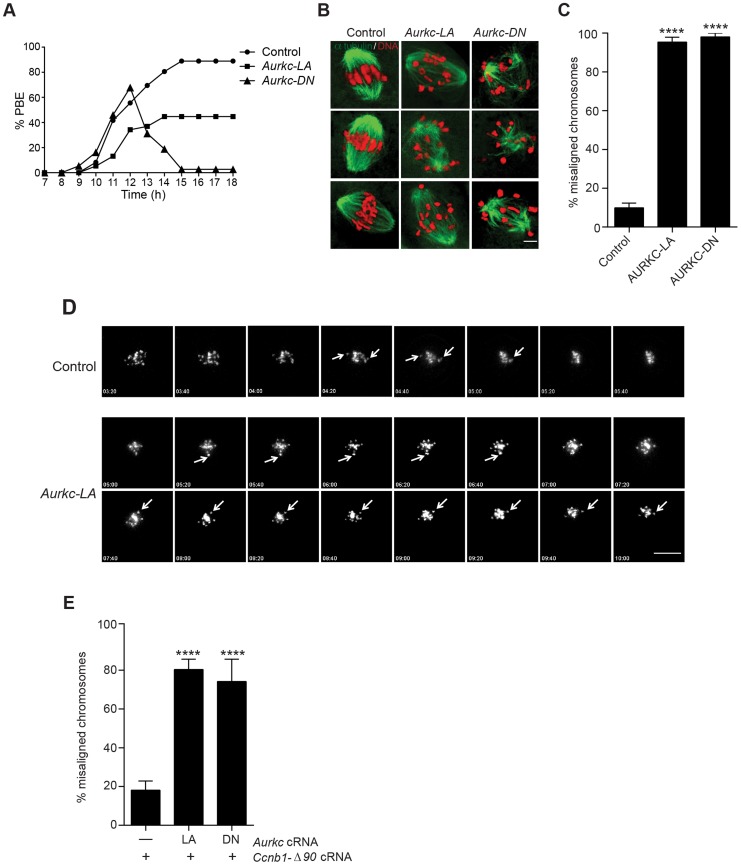
Meiotic progression to Met II and chromosome alignment at Met I requires AURKC. (A) Full-grown WT oocytes from CF1 mice were injected with the indicated cRNA, followed by *in vitro* maturation (16 h) and analysis of the timing of polar body extrusion (PBE) by live cell imaging. The experiment was carried out 2 times with a minimum of 30 oocytes in each group. (B) Representative confocal Z-projections of DNA (red) and spindle configurations (green) from oocytes at Met I (7 h after milrinone washout) that were injected with the indicated cRNA. The experiment was conducted 3 times with a minimum of 30 oocytes in each group (Scale bar, 10 µm). (C) Quantification of the number of oocytes with misaligned chromosomes analyzed in B. (D) Representative H2B-mCherry fluorescence images of oocytes coinjected with the indicated cRNA and *H2B-mCherry* cRNA; the white arrows indicate non-aligned bivalent chromosomes (Scale bar, 50 µm) (E) Met I exit was blocked by microinjection of non-degradable cyclin B (150 ng/µl) mixed with the indicated cRNA, and examined for chromosome alignment by immunocytochemistry. Controls were injected with either PBS or *Gfp* cRNA. The experiment was conducted 2 times with a minimum of 20 oocytes in each group. One-way ANOVA was used to analyze the data. **** P<0.0001.

Similar to AURKC-DN expressing oocytes, oocytes expressing AURKC-LA were defective in meiotic progression. The majority of oocytes expressing AURKC-LA (∼60%) failed to extrude PBs. The kinetics with which those that did extrude PBs were ∼1 h delayed compared to controls. For AURKC-DN expressing oocytes, this failure was more pronounced (∼95%); these oocytes initially extruded PBs, but then retracted them, as previously reported (Figure 6A) [6]. The discrepancy suggests that AURKB carries out meiotic functions during MI that do not require AURKC activity.

To understand the biological significance of this different phenotype, we first focused on Met I. Both AURKC-LA and AURKC-DN expressing oocytes have nearly the same chromosome misalignment phenotype at Met I (Figure 6B–C), suggesting that AURKC-CPC is the main CPC complex from prophase of MI through Met I, and that AURKC is essential for chromosome alignment. To examine the chromosome alignment phenotype in more detail, we imaged control-injected and AURKC-LA-injected oocytes live. Both groups expressed H2B-GFP to mark chromosomes. Unlike in controls, chromosomes in AURKC-LA oocytes oscillated between nearly aligned and misaligned for the duration of the imaging (Figure 6D, Movies S1, S2).

The presence of misaligned chromosomes in oocytes expressing AURKC-LA could be due to a chromosome alignment problem, or may reflect a cell-cycle delay in oocyte progression to Met I. To discriminate between these possibilities, we blocked Met I exit by injecting oocytes with non-degradable cyclin B1 (*Ccnb1*-Δ90) [23], [43] to allow oocytes more time to align their chromosomes. Strikingly, unlike control oocytes, the majority of the oocytes expressing AURKC-LA still had misaligned chromosomes even after spending 8 hours at Met I (Figure 6E). These data indicate that AURKC activity is indispensable for chromosome alignment in mouse oocyte meiosis.

### AURKC-CPC is not the sole CPC complex that regulates the SAC

The majority of oocytes expressing AURKC-LA arrested at Met I with bivalent chromosomes (Figure S1). Given the severe chromosome misalignment at Met I, it was expected that AURK-DN expressing oocytes would also arrest at Met I [44]. As previously reported, all oocytes expressing AURKC-DN contain univalent chromosomes (Figure S1) [6]. The presence of univalents suggests an active Anaphase Promoting Complex/Cyclosome (APC/C) and separation of homologous chromosomes. The regulatory mechanism responsible for controlling APC/C is called the Spindle Assembly Checkpoint (SAC). The SAC signals the delay of anaphase onset until all chromosomes acquire the correct kinetochore-microtubule attachment either in mitosis [45], [46] or oocyte meiosis [9], [10], [47], [48].

**Figure 7:**
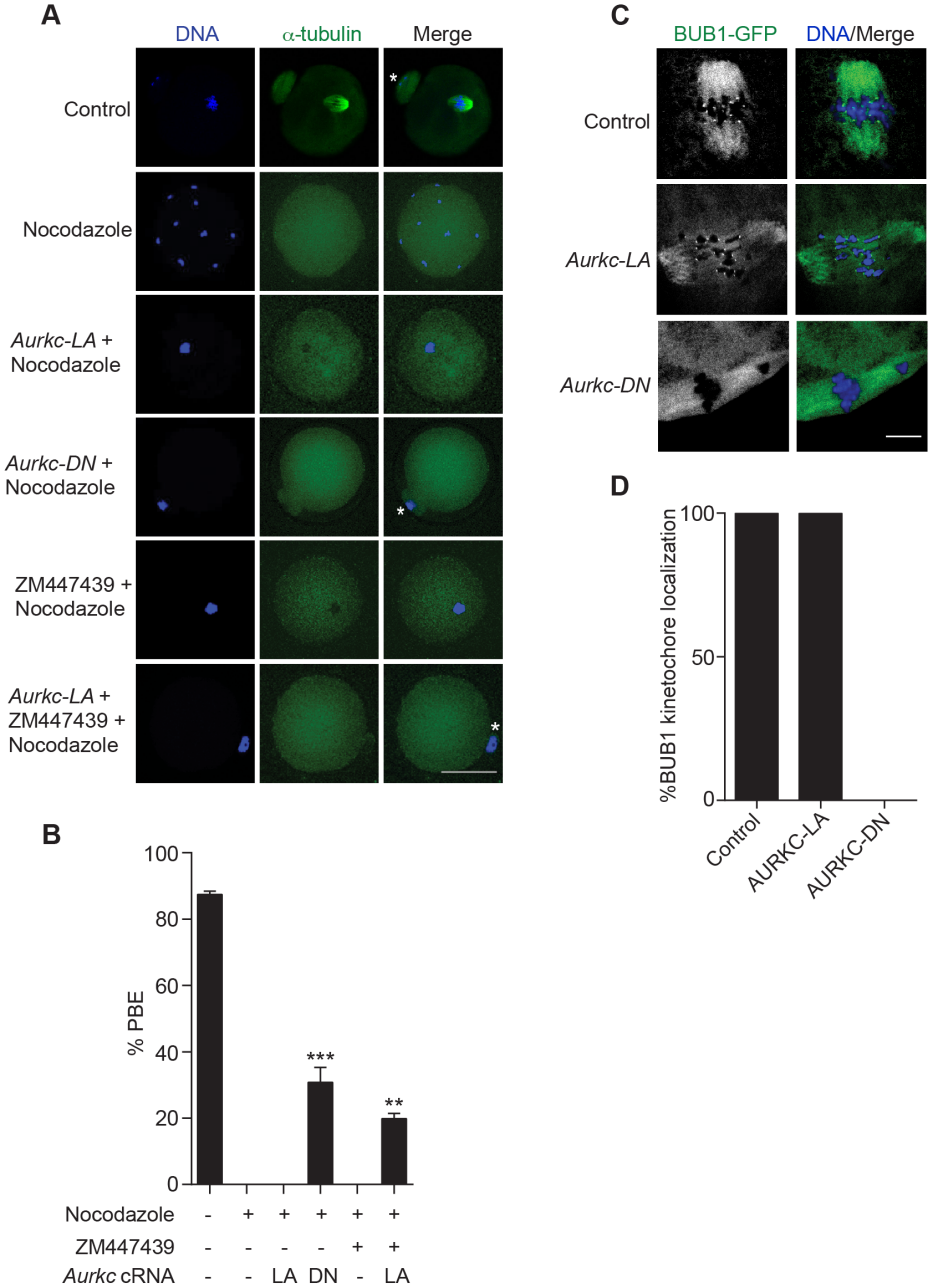
AURKC does not maintain SAC activation by itself. (A) Full-grown oocytes were injected with the indicated cRNA; controls were injected with PBS or *Gfp* cRNA. Nocodazole and ZM447439 were added to the maturation medium as indicated to a final concentration of 5 µM and 2 µM, respectively. After maturation for 16 h, the oocytes were examined for extrusion of the first polar body (PBE) and spindle formation (green) via fluorescence microscopy (scale bar, 50 µm). DNA was detected via DAPI staining (blue). The experiment was conducted 3 times with a minimum of 30 oocytes in each group. Shown are representative images; the white asterisks mark PBs. (B) Quantification of the percentage of oocytes that extrude a polar body (PBE) in A. One-way ANOVA was used to analyze the data. ** P<0.01; *** P<0.001. (C) *Bub1-Gfp* cRNA (300 ng/µl) was co-injected with the indicated cRNA and *in vitro* matured oocytes were then examined by confocal microscopy to detect GFP (green in merge). DNA was detected via DAPI staining (blue). Shown are representative Z-projections (scale bar, 10 µm). The experiment was conducted 2 times with a minimum of 20 oocytes in each group. (D) Quantification of the percentage of oocytes in C that contained BUB1-GFP at kinetochores.

We investigated the ability of AURKC-LA and AURKC-DN to maintain the SAC. To conduct these studies, we incubated control, *Aurkc-LA* or *Aurkc-DN* injected oocytes in nocodazole, a microtubule-depolymerizing drug that keeps the SAC active in WT cells because of an absence of K-MT attachments. As expected only oocytes expressing AURKC-DN extruded PBs in the presence of nocodazole (Figure 7A,B). We obtained similar results when nocodazole was used at a lower dose that does not completely depolymerize the spindle (Figure S3). These results indicate that AURKB has a role in maintaining an active SAC signal.

ZM447439 is a pan-Aurora kinase inhibitor with higher specificity for AURKB than AURKC and AURKA [49]. Oocytes incubated in a high concentration of ZM447439 (10 µM) bypass the SAC [30]. This dose likely inhibits both AURKB and AURKC. Our data indicate that AURKC-CPC is not the sole CPC involved in SAC signaling, but it possible that its function overlaps with AURKB. To investigate this possibility, we incubated oocytes expressing AURKC-LA with a low dose of ZM447439 (2 µM) that does not normally bypass the SAC (Figure 7A–B), in the presence of nocodazole. This is a dose that likely only inhibits AURKB. When AURKB was inhibited in oocytes expressing AURKC-LA, they bypassed the SAC and extruded PBs (Figure 7A,B). These data suggest that the SAC is controlled by both AURKB and AURKC.

In somatic cells, the CPC kinase (AURKB) promotes the kinetochore recruitment of key SAC components including BUB1 (Budding uninhibited by benzimidazoles 1) [50]. To further validate our findings, we microinjected *Bub1-Gfp* cRNA [48] along with *Aurkc-LA* or *Aurkc-DN* cRNAs into oocytes. Again, loss of AURKC function alone did not perturb BUB1 kinetochore localization (Figure 7C–D). But when AURKB was also inhibited, BUB1 failed to localize to the kinetochores (Figure 7C–D). These data confirm that AURKC is not the sole CPC kinase involved in SAC signaling.

### AURKC-CPC is not the sole CPC for cytokinesis

**Figure 8:**
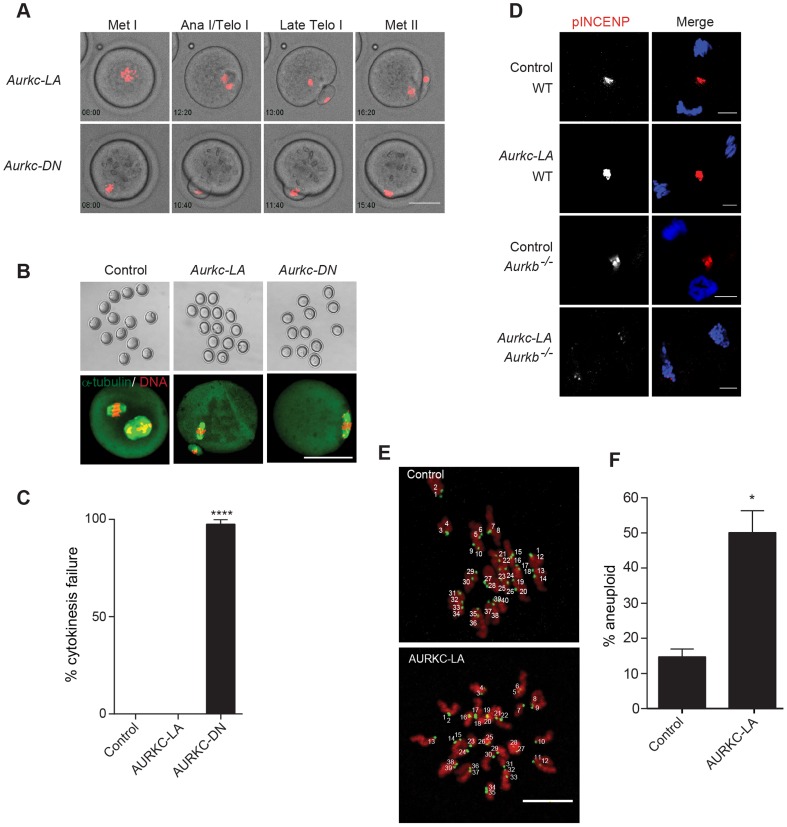
AURKC alone does not regulate cytokinesis and loss of its function leads to aneuploid eggs. (A) Snapshots from a time-lapse series showing chromatin (H2B-mCherry; red) and bright field images from oocytes co-injected with H2B-mCherry and the indicated GFP-tagged cRNA (Scale bar, 50 µm). (B) Representative Z-projections obtained by confocal microscopy of spindle (green) and DNA (red) configurations of Met II eggs (scale bar, 50 µm). (C) Percentage of oocytes that failed cytokinesis. The experiment was conducted 3 times and at least 20 oocytes were examined in each group. (D) WT and *Aurkb^−/−^* oocytes were microinjected with *Aurkc-LA* cRNA followed by maturation to telophase I and examination of phosphorylated INCENP (pINCENP) (red in merge) (scale bar, 10 µm). DNA was detected by DAPI (blue). Shown are representative examples. (E) Met II eggs from the indicated groups were treated with monastrol followed by detection of DNA (red) and kinetochores with Crest anti-sera (green) (scale bar, 10 µm). The number of kinetochores was counted in each egg, and an aberration of 40 was scored as aneuploid. The experiment was conducted 3 times with a minimum of 20 oocytes in each experiment. Shown are representative Z-projections. (F) Quantification of D. One-way ANOVA was used to analyze the data in B and Student's t-test was used to analyze the data in E. Controls were injected with either PBS or *Gfp* cRNA. Values with asterisks vary significantly, *P<0.05; **** P<0.0001.

Arrest at Met I is the predominant phenotype observed in oocytes expressing AURKC-LA, but there is small percentage of oocytes which do extrude PBs (Figure 6A). Consistent with Yang et al., AURKC-DN expressing oocytes began to extrude PBs, but failed to complete cytokinesis and subsequently retracted the PBs [6] (Figure 8A, Movie S4). This phenotype is reminiscent of oocytes cultured in pan Aurora kinase inhibitors ZM447439 and AZD1152 [17], [25], [32]. Unlike AURKC-DN, AURKC-LA expressing oocytes that progressed through Met I extruded PBs normally without any evidence of cytokinesis failure suggesting that AURK-CPC is not the sole CPC controlling cytokinesis, and that AURKB may be important for this function (Figure 8A–C; Movie S3). Progression to Met II did not depend on expression level of the mutant protein. In a zoomed out image of supplemental movie 3 (Movie S5), the oocyte expressing less AURKC-LA arrested at Met I, and the oocyte expressing more AURKC-LA extruded a polar body. To further confirm our hypothesis, we investigated pINCENP as a marker of CPC activity at telophase I (Telo I). Similar to controls, oocytes expressing AURKC-LA contained phosphorylated INCENP at the mid-body (Figure 8D). These data further support our observations that AURKC activity is dispensable for cytokinesis in oocytes. Oocytes lacking AURKB contain pINCENP at the midbody, but when we microinjected *Aurkc-LA* cRNA in *Aurkb* ^−/−^ oocytes (contain only AURKC) we did not detect phosphorylated INCENP. These data suggest overlapping AURKB-CPC and AURKC-CPC activities control cytokinesis (Figure 8D).

### Disruption of AURKC function leads to aneuploid eggs

To investigate the biological significance of selectively perturbing AURKC during MI, AURKC-LA expressing oocytes were examined for aneuploidy using an *in situ* chromosome spread method [7], [51]. The percentage of aneuploid eggs was significantly higher in *Aurkc-LA*-injected oocytes (that did not arrest at Met I) compared to controls (Figure 8E–F). We did not assess ploidy when both AURKB and AURKC kinases were perturbed because no PBs were extruded, and therefore resulted in 100% polyploidy, as previously described [6]. Thus, AURKC function is critical for faithful chromosome segregation in oocyte meiosis.

### AURKC-CPC is the predominant CPC that corrects erroneous K-MT attachments

In mouse oocytes lateral interactions between microtubules and chromosomes drive the early stages of pro-Met I, but the final and sharp alignment of chromosomes at the Met I plate requires end-on K-MT attachments [52]. Brunet and colleagues performed nocodazole washout experiments to examine spindle recovery in mouse oocytes. They found that some chromosomes moved towards the spindle poles (where K-MT end-on attachment is established) before congressing to the metaphase plate. In agreement with this observation, in tissue culture cell lines the mal-oriented, but not bi-oriented, chromosomes move to the mitotic spindle pole until correct attachments are made, and then alignment at the metaphase plate is achieved [53]. We therefore hypothesized that failure to correct erroneous K-MT attachments leads to the misaligned chromosomes that are adjacent to the spindle poles (Figure 6B), in *Aurkc-LA*-injected oocytes.

**Figure 9:**
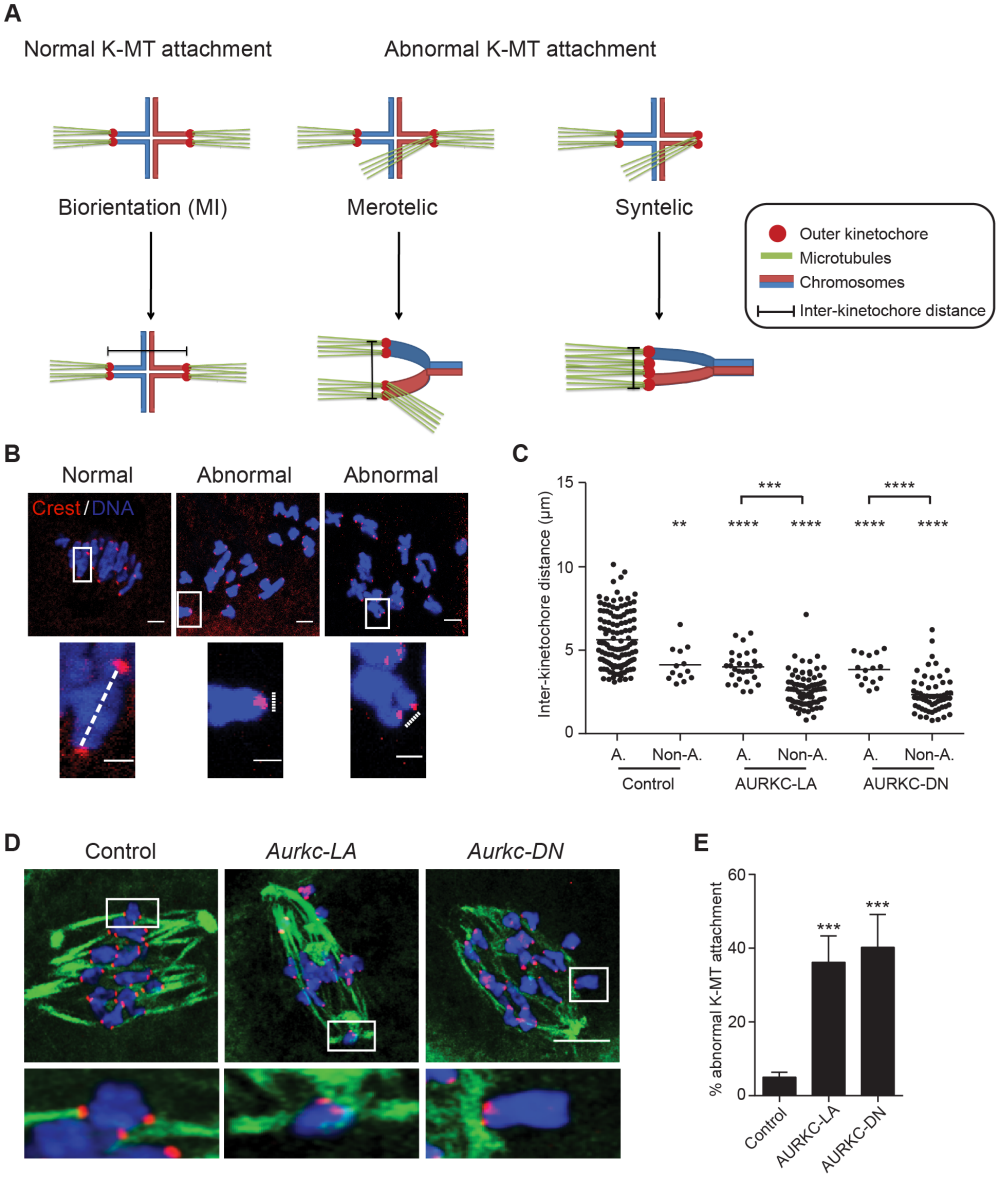
AURKC is the primary CPC kinase that corrects erroneous K-MT attachments. (A) Schematic representation of normal and abnormal K-MT attachments. Sister chromatids are indicated in the same color. Note that chiasmata linking the homologous chromosomes were omitted for simplicity purposes. (B–E) GV oocytes were microinjected with the indicated cRNA and matured to Met I. Kinetochores were labeled with CREST (red) and inter-kinetochore distance was measured using Image J (dotted line). DNA was counterstained with DAPI (blue). The scale bar represents 10 µm for the original images and 2 µm for the magnified images. (C) Quantification of the inter-kinetochore distance from aligned (A.) and misaligned (Non-A.) chromosomes. Each data point is the distance between two sister kinetochore pairs within a bivalent chromosome in an oocyte. The experiment was conducted 3 times with a minimum of 20 oocytes in each group. (D) Representative images of K-MT attachments. Oocytes were incubated in ice-cold medium to depolymerize non-kinetochore attached tubulin prior to fixation and detection of kinetochores (red), tubulin (green) and DNA (blue) (Scale bar, 10 µm). The experiment was conducted 3 times with a minimum of 15 oocytes in each group. (E) Quantification of abnormal K-MT attachments. One-way ANOVA was used to analyze the data. ** P<0.01, *** P<0.001, **** P<0.0001.

When K-MT attachments are correct at Met I, the bivalent chromosomes are bi-oriented with monotelic attachment of each sister pair to opposite poles. This type of attachment generates tension leading to greater separation between the two sister-kinetochore pairs of each homologous chromosome. Incorrect attachment (merotelic and syntelic) leads to decreased tension and reduced separation between the two sister-kinetochore pairs of each homologous chromosome (Figure 9A–B) [10], [54]. Similar to AURKC-DN-expressing oocytes, oocytes expressing AURKC-LA showed significantly shorter inter-kinetochore distance (detected by CREST anti-serum) compared to control oocytes. These data imply that the error correction mechanism is impaired in these oocytes. Moreover, the majority of the misaligned bivalents had incorrect attachments as evidenced by the decrease of the inter-kinetochore distance (Figure 9C). These data suggest that the chromosome misalignment phenotype after disruption of AURKC function might be, at least in part, due to a defect in correcting improper K-MT attachments. This result is consistent with the conclusion that mitotic cells lacking AURKB activity fail to align chromosomes due to inability to correct abnormal attachment [54].

To further confirm that correcting erroneous K-MT attachments depends upon AURKC, we conducted an assay to determine the presence of stable end-on attachments of K-MTs to kinetochores. Microtubules that form stable kinetochore attachment are cold stable, whereas microtubules that do not form stable attachments with kinetochores are cold labile [55]. We exposed Met I oocytes to a pulse of cold medium, prior to fixation and immunocytochemistry to detect kinetochores and microtubules. *Aurkc-LA*-injected oocytes had a significantly greater percentage of abnormal (merotelic and syntelic) attachments than *mCherry*-injected controls (Figure 9D–E). The percentage of abnormal K-MT attachments in *Aurkc-LA*-injected oocytes was similar to that of *Aurkc-DN*-injected oocytes (Figure 9E and [6]). We suggest that AURKC-CPC is the predominant form of the CPC that corrects erroneous K-MT attachments and for chromosome alignment in mouse oocyte meiosis.

## Discussion

Distinguishing the roles of AURKB and C has been complicated by many factors. The two kinases are highly similar in sequence and appear to compensate for one another. One logical interpretation is that AURKB is the predominant CPC kinase in mitosis while AURKC is the predominant CPC kinase in meiosis. This model is supported by a report that detected no AURKB protein in mouse oocytes by immunoblotting [6]. But a collection of observations suggests that AURKB is found in oocytes. For example, oocytes express *Aurkb* mRNA [24], [25] and overexpression of AURKB, but not AURKC, rescues defects induced by a low dose of ZM447439, a pan Aurora kinase inhibitor with highest affinity for AURKB [24], [33], [49]. In this report, using a different antibody and mouse strains than in the previous report [6] we detected AURKB protein in mouse oocytes by immunoblot, and showed that it localized to centromeres in *Aurkc^−/−^* oocytes (Figure 1). These data provide evidence that AURKB is expressed in mouse oocytes and support our previous report [23]. Dominant negative alleles of AURKB and AURKC perturb themselves and one another when expressed in mitosis [22]. It is therefore not surprising that when we expressed AURKC-DN in *Aurkc* ^−/−^ oocytes, endogenous AURKB was also perturbed (Figure 2B). A second model to consider is the notion that the mouse genome may contain multiple copies of *Aurkc* [56] and that the knockout is not completely void of AURKC protein. Another group has revisited the updated mouse genome sequence and found that coding regions of *Aurkc* are not duplicated [57]. Moreover, when we probed oocytes from *Aurkc* ^−/−^ mice, we did not detect any *Aurkc* transcript or protein [23]. Importantly, until this study, whether AURKB and AURKC have any non-overlapping functions was not known.

By selectively disrupting AURKC function in oocytes, we have shown for the first time that AURKC has distinct functions from AURKB in mouse oocytes. We find that AURKC corrects erroneous K-MT attachments, a likely cause of chromosome misalignment at Met I (Figures 6, 9). This failure to align chromosomes caused a Met I arrest, as one would expect given an intact SAC (Figure 7). But the small percentage of oocytes that presumably had mild chromosome misalignment, likely below the threshold of maintaining SAC activation, extruded PBs without any evidence of cytokinesis failure (Figure 8). We found that these oocytes were aneuploid (Figure 8). In our experiments, AURKC-LA is expressed from the GV stage though out meiosis. We note that, this prolonged duration of expression could make analysis of the Met II phenotype more challenging. These later roles of AURKB and AURKC will be important to address in future studies. Thus, AURKC-CPC appears to be the predominant CPC that corrects improper K-MT attachments, a function essential for preventing aneuploidy.

We are interested in understanding why meiosis might require two CPC kinases whereas most mitotic cells have only one. In mitosis, AURKB directly maintains SAC activation by recruiting components such as BUB1 to kinetochores [39], [50], [58], [59] and indirectly participates in the SAC by destabilizing K-MTs. Given the presence of two forms of the CPC in oocyte meiosis, we propose a separation of function model: AURKB-CPC recruits BUB1 to kinetochores, while AURKC-CPC destabilizes improper K-MT attachments. In agreement with this hypothesis, we observed bypass of SAC-inducing conditions only when we inhibited both AURKB/C (Figure 6) and arrest at Met I when we inhibited only AURKC (Figure 6). This strategy to use complementary AURKB and AURKC functions to control the SAC may be critical to provide an insurance mechanism to prevent aneuploidy in a transcriptionally quiescent cell type where AURKB protein is not stable [23].

Loss of AURKC function did not affect pINCENP at the mid-body or induce cytokinesis failure (Figure 8). These data suggest that the CPC containing AURKC as the catalytic subunit is not the predominant form of the CPC that regulates cytokinesis. AURKB-CPC plays an important role in mitotic cytokinesis by phosphorylating many substrates, including INCENP, at the midbody [15], [16], [60], [61]. However, INCENP is phosphorylated in oocytes that lack AURKB (Figure 8). It is possible that AURKC compensates for loss of AURKB in the knockout oocytes. Interestingly, mitotic cytokinesis requires an increased amount of AURKB activity, as compared to its metaphase functions [62]. AURKB and AURKC both localize to the midbody in oocytes [24], [33]. Therefore, it is possible that oocytes satisfy the need for elevated AURK activity at the midbody by having overlapping functions of 2 forms of the CPC available, differing only in the catalytic subunit, and further examination is needed.

We have not yet determined why the gatekeeper mutant displays specificity for affecting only AURKC. We have eliminated that possibility that these mutants were expressed at different levels in our system or in the different genetic backgrounds (Figure S4). We have also ruled out possible differences in catalytic activity because oocytes expressing AURKC-LA also showed complete loss of AURKC and INCENP phosphorylation (Figure 3). In both mutants the activation loop is not phosphorylated but the proteins are different. In the DN protein, the threonines are mutated to alanines, whereas in the LA protein the threonines are present but do not contain phosphate. The activation loop of protein kinases is important not only for catalytic activity but also for conformation stabilization, the ability to bind substrates, and for substrate specificity [63]. The conformation of the ATP binding pocket is also critical for protein structure [22]. Similar to our observations with AURKC-DN, mutation of the activation loop threonines to alanines in protein kinase C (PKC) alpha loosens its specificity and the mutant inhibits the other PKC isoforms [64]. Although we are not certain as to the mechanism of inhibition of the LA protein, one model to investigate is that AURKC functions as a dimer within the CPC, and that the gatekeeper mutant functions as a dominant negative only in the context of an AURKC dimer. To our knowledge there is no evidence that AURKB dimerizes, and it would be interesting if this mechanism were AURKC-specific. Alternatively, AURKC-LA may function as a pseudokinase. Pseudokinases have high sequence homology to kinases but do not have detectable catalytic activity [65], [66]. Some of these proteins contain amino acid substitutions in gatekeeper residues of their ATP binding pockets that would ablate ATP binding or efficient catalysis [65]. If AURKC-LA were acting as a pseudokinase it could be preventing WT AURKC from binding the CPC. Most significantly, in *Aurkc^−/−^* oocytes, where AURKB is the only CPC kinase, expression of AURKC-LA but not AURKC-DN resulted in normal meiotic progression and CPC kinase activity. Therefore it is clear that the main difference between these mutants is the inability of AURKC-LA to compete with endogenous AURKB function.

To our knowledge, this is the first report to separate AURKB and AURKC meiotic functions, and is consistent with some of the proposed models [17], [23]. AURKC is expressed in other cell types, including testes, neuronal tissue and some cancer cells [21], [29], [67], [68]. The biological significance of AURKC expression in cancer cells is also of clinical interest, but not well understood. Because these cells also express AURKB, studying the functions of AURKC in cancer cell division poses the same specficitiy difficulties as in oocytes. With our validation of AURKC-LA being specific for disrupting AURKC function, we propose that this gatekeeper mutant will be helpful tool for answering questions relevant to the reproductive and cancer fields

## Materials and Methods

### Generation and genotyping of *Aurkc^−/−^* mice and *Aurkb^fl/fl^ ZP3-Cre* mice

Details for generating and genotyping *Aurkc* ^−/−^ mice were described previously [23], [67]. The *Aurkb^fl/fl^* mice were a generous gift from M. Malumbres (CNIO, Spain) [29]. For generating *Aurkb* ^fl/fl^ *ZP3-Cre* mice, female mice carrying the *Aurkb* floxed alleles were crossed with ZP3-Cre males (Jackson laboratories) [69], and genotyping for the LoxP sites was carried out as previously described [29]. Cre genotyping was carried out as described by Jackson Laboratories. A detailed phenotypic description will be described elsewhere. All animals were in a mixed background of C57BL/6J, 129/Sv, and CD1 and maintained following Institutional Animal Use and Care Committee and National Institutes of Health (NIH) guidelines.

### Cloning, mutagenesis and *in vitro* cRNA synthesis

Generation of non-degradable *cyclin B*, *Aurka*, *Aurkb*, and *Aurkc*-*Gfp* were described previously [24], [43]. To generate *Bub1*-*Gfp*, murine *Bub*, sequence was amplified via PCR from a cDNA clone, (Open Biosystems, #3671932) and ligated into pIVT-GFP [70]. *Aurkc*-*LA* and *Aurkc*-*DN* mutants were generated by site-directed mutagenesis using the QuikChange Multi-site Mutagenesis kit (Agilent Technologies) following manufacturer's instructions. To generate *Aurkc*-*DN* T171 and 175 were changed to an A (ACA and ACT to GCC; Figure 2A). To generate *Aurkc*-L93 was changed to an A (CTG to GCC; Figure 3A).

DNA linearization of all *Gfp*- and *mCherry*- containing constructs was carried out using Nde I (New England BioLabs). After DNA linearization, the digests were purified (Qiagen, QIAquick PCR Purification) and *in vitro* transcription was carried out using an mMessage mMachine T7 kit (Ambion) according to the manufacturer's instructions. Finally, the cRNA was purified using an RNAEasy kit (Qiagen).

### Oocyte collection, microinjection and culture

Full-grown, GV-intact oocytes were obtained from pregnant mare serum gonadotropin- (PMSG) (Calbiochem #367222) primed (44–48 h before collection), 6-wk-old female mice as previously described [71]. The collection and injection medium for oocytes was bicarbonate-free minimal essential medium (MEM) containing, 25 mM Hepes, pH 7.3, 3 mg/ml polyvinylpyrollidone (MEM/PVP) and 2.5 µM milrinone (Sigma #M4659) to prevent meiotic resumption [72].

Denuded GV oocytes were microinjected with ∼10 pl of 0.8–1 µg/µl of the indicated cRNA, unless otherwise noted. Following microinjection, the oocytes were cultured in Chatot, Ziomek, and Bavister (CZB) medium containing 2.5 µM milrinone. All culture and *in vitro* meiotic maturation occurred in a humidified incubator with 5% CO_2_ in air at 37°C. For the oocytes that were examined at Met II, we incubated the injected oocytes for 1–3 h prior to meiotic maturation, and for the oocytes that were examined at Met I, we incubated the injected oocytes overnight (14 h) prior to meiotic maturation. *In vitro* meiotic maturation was conducted in milrinone-free CZB medium for periods of 6–7 h (Met I), 9 h (Telo I) or 16 h (Met II).

Nocodazole (Sigma #M1404) and ZM447439 (Tocris #2458) were dissolved in dimethyl sulfoxide (DMSO). Nocodazole and ZM447439 were added to CZB culture medium to a final concentration of 5 µM and 2 µM, respectively, and *in vitro* maturation was performed in a humidified chamber (Becton Dickinson #353037).

### Immunocytochemistry and confocal microscopy

For analysis of cold-stable microtubules, oocytes were incubated for 5 minutes on ice in MEM/PVP, and then fixed for 25 minutes at 37°C in 3.7% formaldehyde in 100 mM Pipes, pH 6.8, containing 10 mM EGTA, 1 mM MgCl_2_ and 0.2% Triton X-100 [73]. AURKC-GFP was detected after fixation in 3.7% paraformaldehyde in phosphate-buffered saline (PBS) for 1 hour; survivin was detected by similar fixation conditions plus 0.1% Triton X-100. In all other experiments, oocytes were fixed in 2–2.5% paraformaldehyde in PBS for 20 minutes at room temperature. After fixation, the cells were permeabilized with 0.1% Triton X-100 in PBS for 15 minutes and transferred to blocking buffer (PBS+0.3% BSA+0.01% Tween-20) for 15 minutes. Immunostaining was performed by incubating the fixed oocytes with the primary antibody for 1 hour. After washing in blocking solution, the oocytes were incubated in secondary antibodies for 1 hour; omission of the primary antibody served as negative control. DNA was stained and mounted with 4′, 6-Diamidino-2-Phenylindole, Dihydrochloride (DAPI; Life Technologies #D1306; 1∶170) diluted in VectaShield (Vector Laboratories) under a coverslip with gentle compression. Fluorescence was detected on Zeiss 510 Meta laser-scanning confocal microscope under a 63× objective.

All oocytes in the same experiment were processed at the same time. The laser power was adjusted to a level where signal intensity was just below saturation for the group that displayed the highest intensity and all images were then scanned at that pre-determined laser power. The intensity of fluorescence was quantified with NIH image J software keeping the processing parameters identical when experimental analysis required intensity measurements.

### Live cell imaging

Oocytes microinjected with the indicated cRNAs and histone H2B-mCherry cRNA were transferred into separate drops of CZB medium covered with mineral oil in a 96 well dish (Greiner Bio One, #655892). Bright field, GFP and mCherry image acquisition was started at the GV stage using an EVOS FL Auto Imaging System (Life Technologies) with a 20× objective. The microscope stage was heated to 37°C and 5% CO_2_ was maintained using the EVOS Onstage Incubator. Images of individual cells were acquired every 20 min and processed using NIH image J software.

### Antibodies

The following primary antibodies were used in immunofluorescence: CREST autoimmune serum (Antibodies Incorporated; #15-234; 1∶30), AURKB (Abcam #AB2254; 1∶50), AURKC (Bethyl #A400-023A- BL1217; 1∶30), pAURKC (kind gift of T. Tang, Institute of Biomedical Science, Taiwan [6]; 1∶500), phospho-specific Ser893/Ser894 INCENP (kind gift of M. Lampson, UPenn [74]; 1∶1,000), survivin (Cell Signaling Technology #2808S; 1∶500), α-tubulin-Alexa Fluor 488 conjugate (Life Technologies #322588; 1∶100), phospho-specific H3S10 (Millipore; #05-806;1∶100).

### *In situ* chromosome counting

Monastrol treatment, immunocytochemical detection of kinetochores and chromosome counting were performed as previously described [75]. Briefly, eggs were cultured for 2 hours in CZB containing 100 µM monastrol (Sigma) to disperse the chromosomes by collapsing the bipolar spindle to a monopolar spindle. Eggs were fixed in freshly prepared 2% paraformaldehyde and stained with CREST anti-serum to detect kinetochores and DAPI to detect DNA. Images were collected at 0.6-µm Z-intervals to capture the entire region of the MII spindle (16–20 µm total). To obtain a chromosome count for each egg, serial confocal sections were analyzed to determine the total number of kinetochores and calculated using NIH image J software.

### Immunoblotting

Oocytes were lysed in 1% SDS, 1% β-mercaptoethanol, 20% glycerol, and 50 mM Tris–HCl (pH 6.8), and denatured at 95°C for 5 min. Proteins separated by electrophoresis in 10% SDS polyacrylamide precast gel. Stained proteins of known molecular mass (range: 14–200 kDa) were run simultaneously as standards. The electrophoretically separated polypeptides were transferred to nitrocellulose membranes using a Trans-Blot Turbo Transfer System (Bio-Rad) then blocked by incubation in 2% blocking (ECL blocking; Amersham) solution in TBS-T (Tris-buffered saline with 0.1% Tween 20) for 1 h. The membranes were then incubated with primary antibodies at 4°C overnight (GFP (Sigma #G6539; 1∶1,000), β actin (Abcam #ab20272; 1∶10,000), AURKB (Abcam #ab2254; 1∶500), α-tubulin (Sigma #T-6074; 1∶10,000). After washing with TBS-T five times, the membranes were incubated with a secondary antibody labeled with horseradish peroxidase for 1 h followed with washing with TBS-T five times. The signals were detected using the ECL Select Western blotting detection reagents (Amersham) following the manufacturer's protocol.

### Statistical analysis

One-way ANOVA and Student's t-test, as indicated in figure legends, were used to evaluate the differences between groups using GraphPad Prism. The differences of p<0.05 were considered significant.

## Supporting Information

Figure S1 Oocytes expressing AURKC-DN but not those expressing AURKC-LA have univalent chromosomes at Met I. Full-grown oocytes were injected with the indicated cRNA; controls were injected with PBS or *Gfp* cRNA. The microinjected oocytes were matured *in vitro* to Met II (16 h). Oocytes that failed to extrude a polar body (Met I-arrested) were fixed and stained with DAPI to detect DNA. The experiment was conducted 3 times with a minimum of 20 oocytes in each group. Shown are representative confocal Z-projections. The scale bars are 10 µm (original images) and 2 µm (magnified images).
(TIF)

Figure S2 AURKB-GFP co-localizes with Survivin in oocytes from *Aurkc* ^−/−^ mice. Full-grown oocytes from WT, *Aurkb* ^−/−^ or *Aurkc* ^−/−^ mice were injected with the indicated cRNA and matured to Met I (8 h) prior to fixation and detection of Survivin. The GFP (green), Survivin (red), and DNA (DAPI; blue) signals were detected by confocal microscopy. Shown are representative confocal Z-projections. The scale bars are 10 µm.
(TIF)

Figure S3 AURKC is not solely required to maintain SAC activation. Full-grown oocytes were injected with the indicated cRNA; controls were injected with PBS or *Gfp* cRNA. Nocodazole and ZM447439 were added to the maturation medium as indicated to a final concentration of 400 nM and 2 µM, respectively. After maturation for 16 h, the oocytes were examined for polar body extrusion (PBE) via confocal microscopy. The experiment was conducted 2 times with a minimum of 30 oocytes in each group. One-way ANOVA was used to analyze the data. ** P<0.01.
(TIF)

Figure S4 The expression levels of AURKC-LA and AURKC-DN are similar. Full-grown oocytes from mice of the indicated genetic background were injected with the indicated *Aurkc* cRNA. After 16 h, 20 Met II oocytes were lysed for immunoblot analysis using an anti-GFP antibody. α-tubulin was used as a loading control and the relative expression levels after normalization to tubulin is indicated in the lower panel.
(TIF)

Movie S1 Chromosomes aligned in control oocyte. Time-lapse microscopic analysis of a living oocyte expressing *H2B-mCherry* (red). Imaging of meiotic maturation began at pro-metaphase I. Time in hours and minutes (h: min) is shown. Acquisitions were taken every 20 min. Scale bar represents 50 µm.
(AVI)

Movie S2 Oocyte expressing AURKC-LA failed to align chromosomes. Time-lapse microscopic analysis of a living oocyte expressing AURKC-LA and H2B-mCherry (red). Imaging of meiotic maturation began at pro-metaphase I. Time in hours and minutes (h: min) is shown. Acquisitions were taken every 20 min. Scale bar represents 50 µm.
(AVI)

Movie S3 AURKC deficiency did not perturb cytokinesis in MI. Time-lapse microscopic analysis of a living oocyte expressing AURKC-LA-GFP (green) and H2B-mCherry (red). Imaging of meiotic maturation began at breakdown of the nuclear envelope. Time in hours and minutes (h: min) is shown. Acquisitions were taken every 20 min. Scale bar represents 50 µm.
(AVI)

Movie S4 Oocyte expressing AURKC-DN failed cytokinesis. Time-lapse microscopic analysis of a living oocyte that expresses AURKC-DN-GFP (green) and H2B-mCherry (red). Imaging of meiotic maturation began at breakdown of the nuclear envelope. Time in hours and minutes (h: min) is shown. Acquisitions were taken every 20 min. Scale bar represents 50 µm.
(AVI)

Movie S5 Zoomed out video of movie S3. The oocyte on the left expresses less *Aurkc-LA-Gfp* than the oocyte on the right. The oocyte on the right was featured in movie S3. Acquisitions were taken every 20 min. Scale bar represents 50 µm.
(AVI)
