# Supplementary figures and images for: Selective Disruption of Aurora C Kinase Reveals Distinct Functions from Aurora B Kinase during Meiosis in Mouse Oocytes
Source: PLoS Genet. 2014 Feb 27;10(2):e1004194. doi: 10.1371/journal.pgen.1004194 (PMC3937256; doi:10.1371/journal.pgen.1004194)

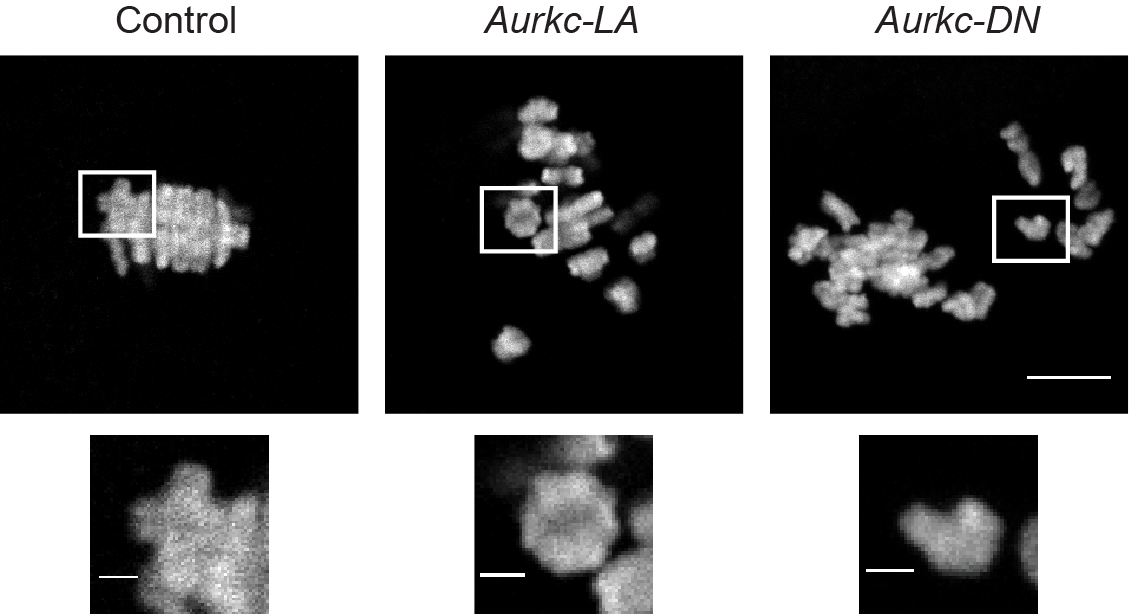

Supplement: Figure S1 — Oocytes expressing AURKC-DN but not those expressing AURKC-LA have univalent chromosomes at Met I. Full-grown oocytes were injected with the indicated cRNA; controls were injected with PBS or Gfp cRNA. The microinjected oocytes were matured in vitro to Met II (16 h). Oocytes that failed to extrude a polar body (Met I-arrested) were fixed and stained with DAPI to detect DNA. The experiment was conducted 3 times with a minimum of 20 oocytes in each group. Shown are representative confocal Z-projections. The scale bars are 10 µm (original images) and 2 µm (magnified images). (TIF) [file pgen.1004194.s001.tif]

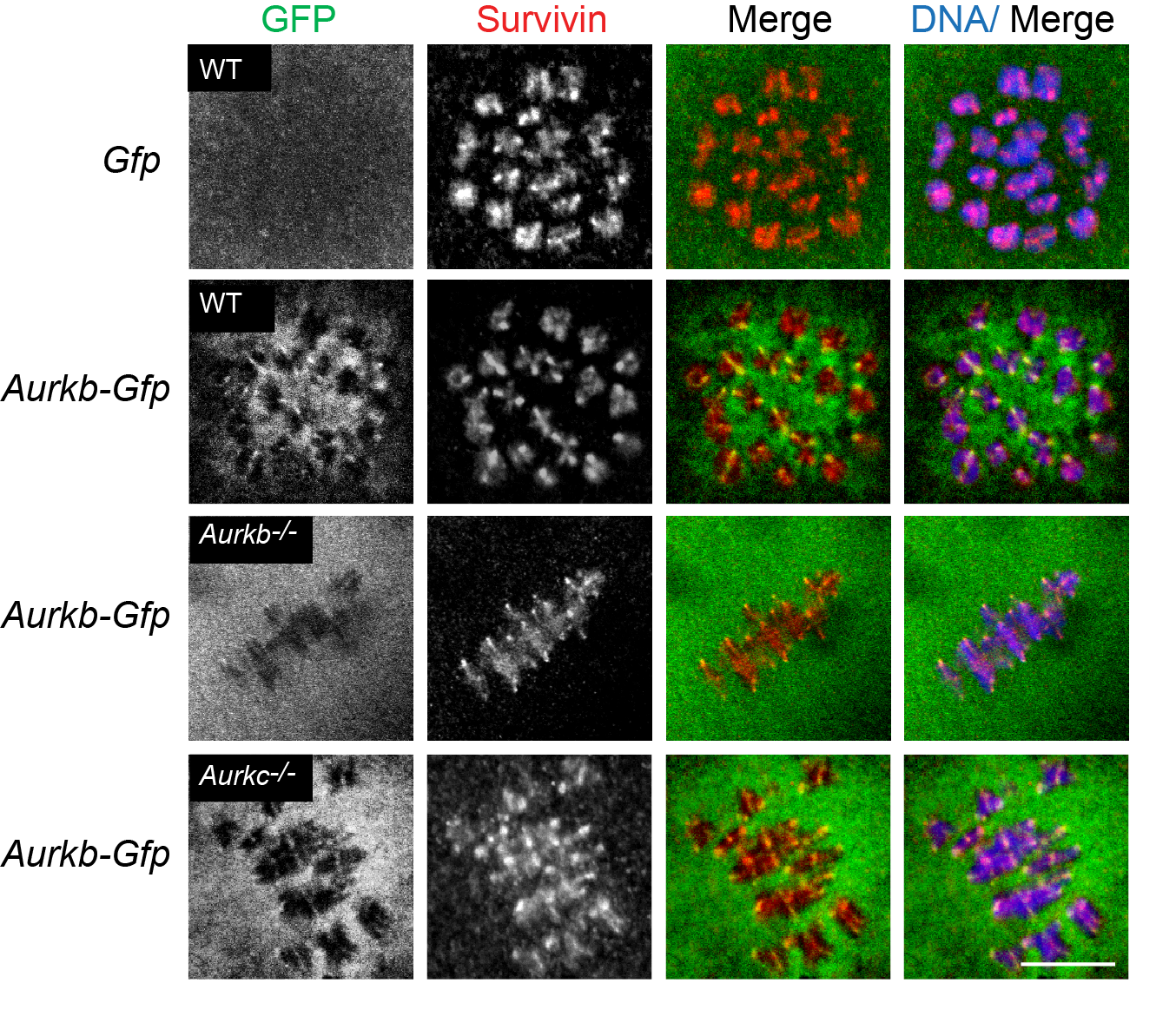

Supplement: Figure S2 — AURKB-GFP co-localizes with Survivin in oocytes from Aurkc −/− mice. Full-grown oocytes from WT, Aurkb −/− or Aurkc −/− mice were injected with the indicated cRNA and matured to Met I (8 h) prior to fixation and detection of Survivin. The GFP (green), Survivin (red), and DNA (DAPI; blue) signals were detected by confocal microscopy. Shown are representative confocal Z-projections. The scale bars are 10 µm. (TIF) [file pgen.1004194.s002.tif]

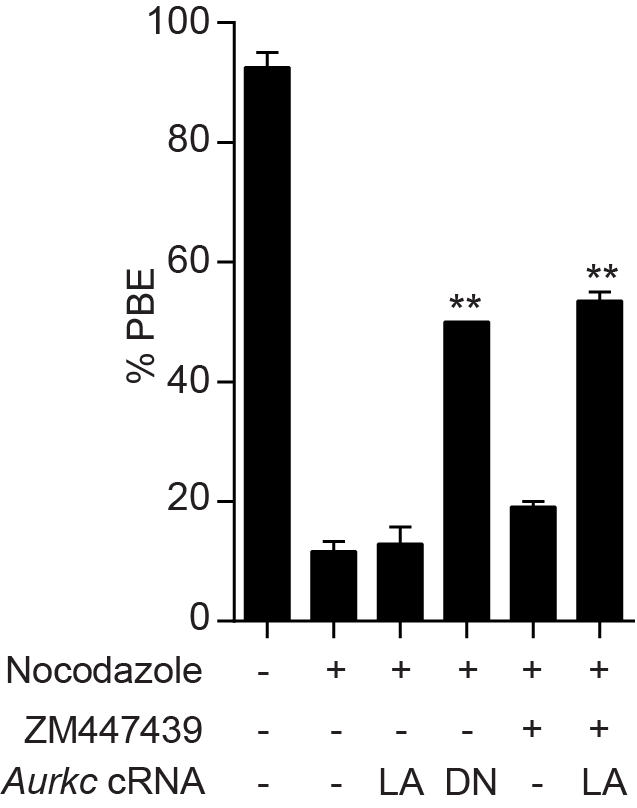

Supplement: Figure S3 — AURKC is not solely required to maintain SAC activation. Full-grown oocytes were injected with the indicated cRNA; controls were injected with PBS or Gfp cRNA. Nocodazole and ZM447439 were added to the maturation medium as indicated to a final concentration of 400 nM and 2 µM, respectively. After maturation for 16 h, the oocytes were examined for polar body extrusion (PBE) via confocal microscopy. The experiment was conducted 2 times with a minimum of 30 oocytes in each group. One-way ANOVA was used to analyze the data. ** P<0.01. (TIF) [file pgen.1004194.s003.tif]

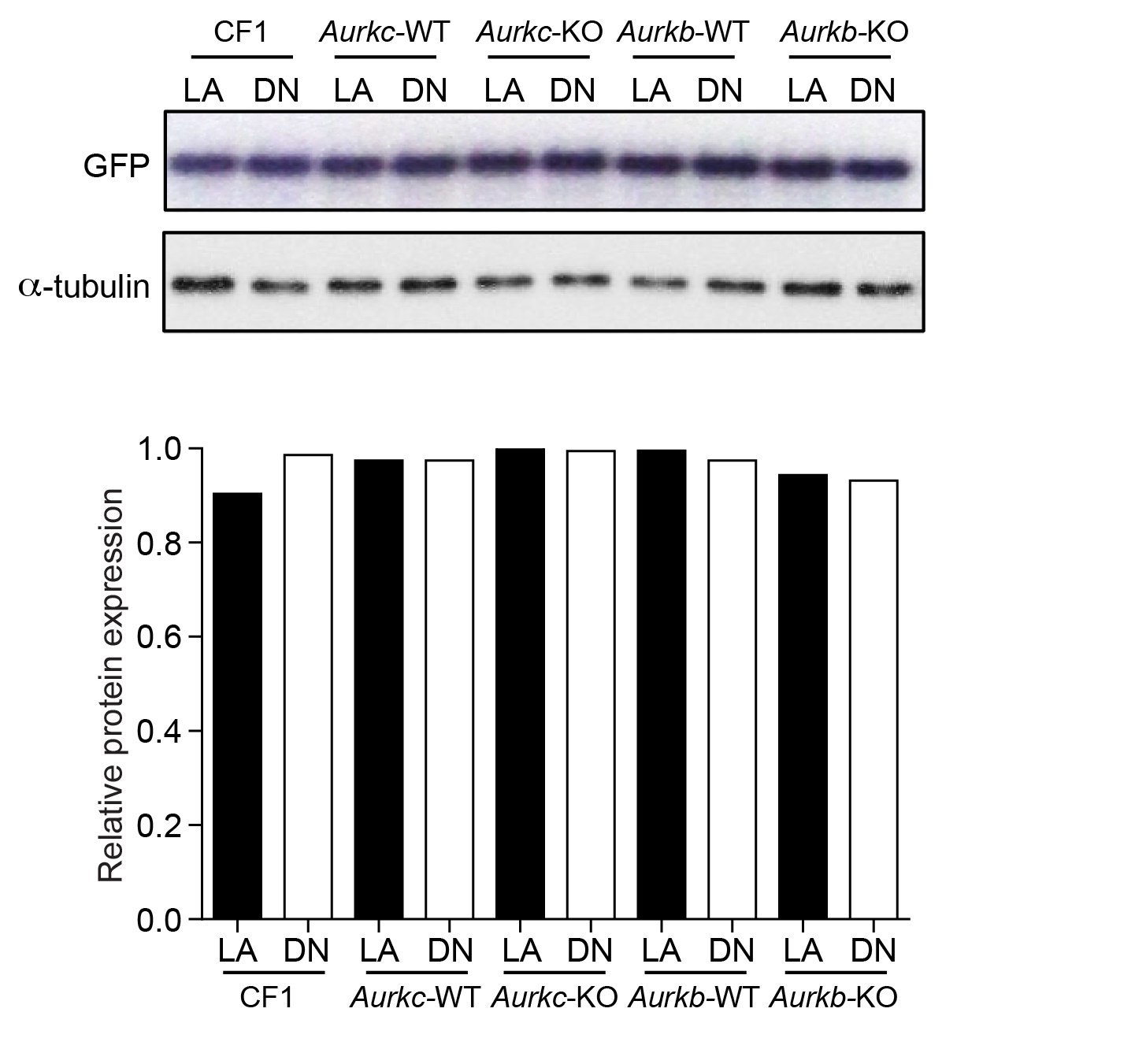

Supplement: Figure S4 — The expression levels of AURKC-LA and AURKC-DN are similar. Full-grown oocytes from mice of the indicated genetic background were injected with the indicated Aurkc cRNA. After 16 h, 20 Met II oocytes were lysed for immunoblot analysis using an anti-GFP antibody. α-tubulin was used as a loading control and the relative expression levels after normalization to tubulin is indicated in the lower panel. (TIF) [file pgen.1004194.s004.tif]
